# Primordial Follicle Response to Two Methods of Ovarian Cortex Retrieval and Vitrification: A Pilot Study

**DOI:** 10.7759/cureus.84918

**Published:** 2025-05-27

**Authors:** Rebeca Chávez-Genaro, Gabriel Anesetti, Lorena Bonjour, Clara Fernández, Agustina Toledo, Karina Hernández, Natalibeth Barrera, Lidia Cantú, Dana Kimelman

**Affiliations:** 1 Histology and Embryology, Universidad de la República, Montevideo, URY; 2 Embryology, Centro de Esterilidad Montevideo, Montevideo, URY; 3 Gynecology, Centro Hospitalario Pereira Rossell, Administración de los Servicios de Salud del Estado, Montevideo, URY

**Keywords:** cryopreservation, histology, oncofertility, ovary, primordial follicles

## Abstract

Cryopreservation and transplantation of ovarian cortical tissue are novel techniques to preserve fertility in young patients undergoing gonadotoxic treatments that may affect fertility. Vitrification has demonstrated growing success in restoring ovarian function and achieving pregnancy post-grafting. It helps maintain communication between follicles and interstitial tissue, which is essential for follicular growth. This study compares two ovarian cortex dissection techniques (strips and layers) using an ovine animal model. The results indicate that manipulation of the ovarian cortex affects primordial follicle activation and stromal tissue during vitrification, potentially compromising oocyte viability and reproductive potential. Additionally, the distribution of primordial follicles in ovarian tissue varies, influencing transplantation efficiency. Both dissection methods increase follicle activation, suggesting that mechanical manipulation impacts outcomes. These findings underscore the need to optimize tissue processing to enhance fertility preservation, particularly by understanding the roles of the stromal and extracellular matrix (ECM) in maintaining follicle dormancy and viability post-vitrification and reimplantation.

## Introduction

The primordial follicle pool represents the entire nonrenewable ovarian reserve that a female mammal will ever possess and that determines their reproductive lifespan [1]. In female cancer patients, the ovarian follicular reserve may be seriously damaged after chemo or radiotherapy treatments, causing endocrine and reproductive function alterations and even infertility [2]. In recent years, the techniques of cryopreservation and transplantation of ovarian tissue have been proven as promising procedures to safeguard and restore the fertility of young patients in whom hormonal stimulation and cryopreservation of mature oocytes or early embryos is not possible [3]. Ovarian tissue vitrification maintains primordial follicles communicated with the interstitial tissue contributing to securing the essential environment for follicular growth and oocyte maturation [4,5]; however, its standardization protocol continues developing [6]. 

Several molecular factors have been identified as candidates for activating dormant primordial follicles into primary follicles. Among these, the PI3K-Akt-Foxo3a pathway stands out as one of the most relevant pathways in this process [7,8]. The activation of PI3K phosphorylates the transcription factor Foxo3a, causing its movement from the nucleus to the cytoplasm, losing its ability to control gene expression and triggering oocyte growth. Inhibition of the PI3K pathway mediated by PTEN signaling is required to maintain primordial follicles at the resting stage [9].

On the other hand, various reports have indicated that intrinsic mechanical tissue pressure imposed by the extracellular matrix (ECM) plays a role in maintaining primordial follicles or promoting their activation within the superficial ovarian cortex [5,10,11]. The interstitial ovarian compartment contains adhesion proteins, extracellular fibers, and cellular components that interact with the follicular unit and contribute to regulating these processes [12].

Preserving both components (primordial follicles and interstitial tissue) during manipulation and conservation of ovarian cortical tissue is not just a technical requirement but a crucial step to ensure adequate restoration of reproductive function after reimplantation. In this paper, we compare the effects of two procedures for ovarian cortex dissection on the histological characteristics of primordial follicles and surrounding interstitial tissue after vitrification and culture using an animal model, which could affect clinical outcomes or protocol standardization, underscoring the importance of our research in the field of reproductive medicine and oncofertility.

## Materials and methods

Collection of biological samples

**Figure 1:**
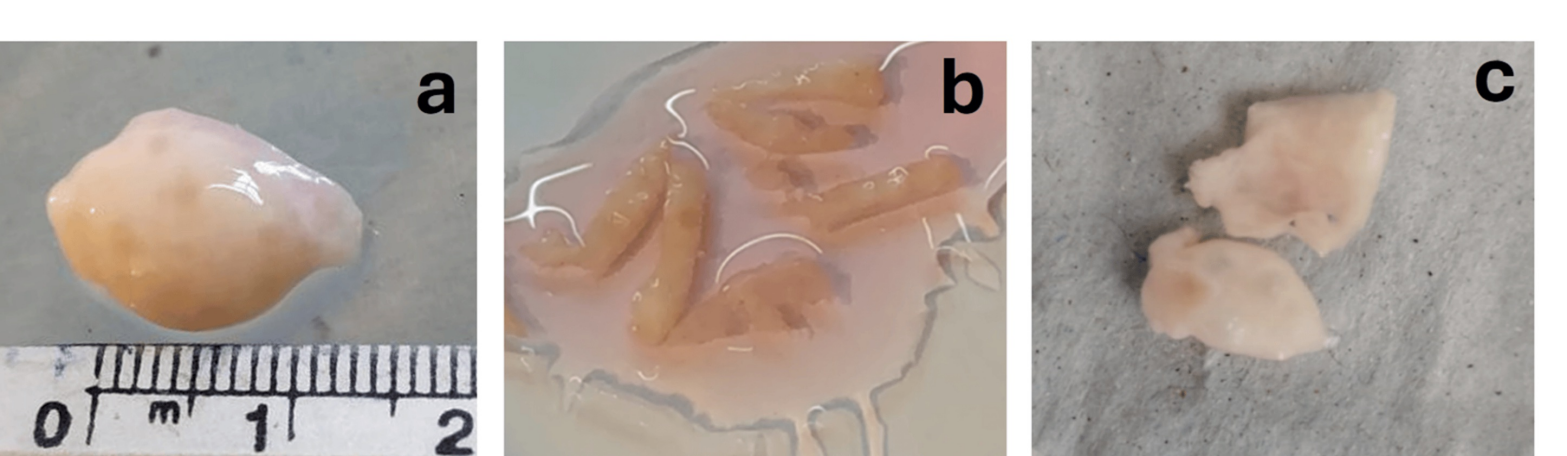
Appearance and size of the ovary (a); tissue cut into strips (b) and layers (c).

Taking into account the similarities between ovine and human ovary architecture, a total of 40 prepubertal sheep ovaries (<1.5 cm, Figure 1a) were obtained from the San Jacinto slaughterhouse (location G537+Q6F, 91600 San Jacinto, Canelones, Department, Uruguay), from July to September 2021. On each visit to the slaughterhouse, two ovaries were fixed in situ in 4% paraformaldehyde (PFA) in phosphate-buffered saline solution (PBS) and were considered as group control (CS). The remaining ovaries were placed in a saline solution containing antibiotics (penicillin-streptomycin) and transported to the laboratory at 4°C within one hour. Upon arrival at the laboratory, fresh ovaries were assigned to dissectors, who processed them using two methods: (a) sectioning the ovary longitudinally through the middle, followed by scraping off the medulla and stromal tissues using fine scissors to obtain a thin layer of cortex, which was immediately cut into strips (12 × 3 × 2 mm: length, width, depth; Figure 1b); or (b) directly cutting the ovarian surface using a plastic device and a fine, sharp blade to obtain superficial layers of tissue (10 × 10 × 2 mm: length, width, depth; Figure 1c). After dissection, all biological samples (strips and layers) were maintained in a culture medium (DMEM, Gibco) until further processing.

Vitrification protocol

**Figure 2:**
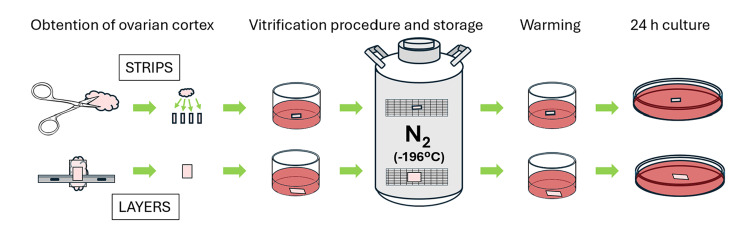
Diagram of the protocol used. Image Credit: Authors (created using PowerPoint software)

After dissection, both strips and layers were vitrified according to protocols described by Laronda et al. [13] and Díaz et al. [14]; briefly, the tissue was placed for 15 minutes in an equilibrium solution and 10 minutes in a thawing solution. Immediately afterward, it was collocated on a metallic mesh, covered with a thin plastic bag, sealed, identified, and stored at -196°C in liquid nitrogen. After two hours, plastic bags were rapidly removed from liquid nitrogen, and the tissue was incubated for three minutes in decreasing concentrations of sucrose solutions. Strips and layers of dissected or vitrified-thawed tissue were transferred to individual culture pits with 1 mL of DMEM supplemented with Insulin-Transferrin-Selenium Liquid Media Supplement (ITS, Sigma) and cultured at 37ºC with 5% CO_2_ for 20 h (Figure 2).

Histological procedure

Samples obtained at the slaughterhouse (CS), at the end of dissection (CD), immediately after vitrification and warming processes (VW), or at the end of the culture period (VWC) were fixed in 4% PFA solution, embedded in paraffin, and processed for the preparation of histological sections (5 µm) for morphological or immunohistochemical evaluation [15]. Sections were deparaffinized, stained with hematoxylin and eosin, and coded for stromal area analysis, measurement of follicular diameter, and identification of the accompanying follicular cell type (flat or cuboidal) by a blinded observer. Another set of histological sections was used for immunohistochemical detection of molecules associated with follicular activation (Foxo3a) or cell death processes (cleaved caspase3). Briefly, hydrated sections were washed in PBS and blocked in a solution containing 2% bovine serum albumin (BSA) and 0.2% Triton-X 100. After that, sections were incubated overnight with primary antibodies diluted in a blocking solution at 4ºC. Next, sections were washed in PBS, sequentially incubated with secondary biotinylated antibodies, streptavidin-HRP, and revealed using diaminobenzidine as the chromogen. Afterward, sections were lightly stained with hematoxylin, dehydrated in increasing ethanol concentrations, cleared in xylol, and coverslipped with DPX. Primary antibodies used were anti-Foxo3a and anti-cleaved caspase3 rabbit monoclonal antibodies (Cell Signaling Technology) at 1:500 dilution. Negative controls omitting primary antibodies were incorporated with each immunohistochemical detection.

Stromal area and collagen fiber quantification

Fiji software and the Trainable Weka Segmentation plug-in were used to identify and measure ovarian stroma areas in H&E-stained histological sections. Similarly, collagen fiber quantification was done using Van Gieson trichrome staining to differentiate collagen fibers in the samples. A blind observer performed the analysis to ensure unbiased results. In both cases, a 300 x 300 μm area, with a magnification of 400x, was randomly selected for image processing. The area occupied by each component was expressed as a percentage.

Statistical analysis

The data were grouped by treatments, and ANOVA or Kruskal-Walli’s test was used to compare groups using GraphPad Prism 9 software. The chi-square test was used to determine differences in percentages between groups. A p < 0.05 was considered significant.

## Results

**Figure 3:**
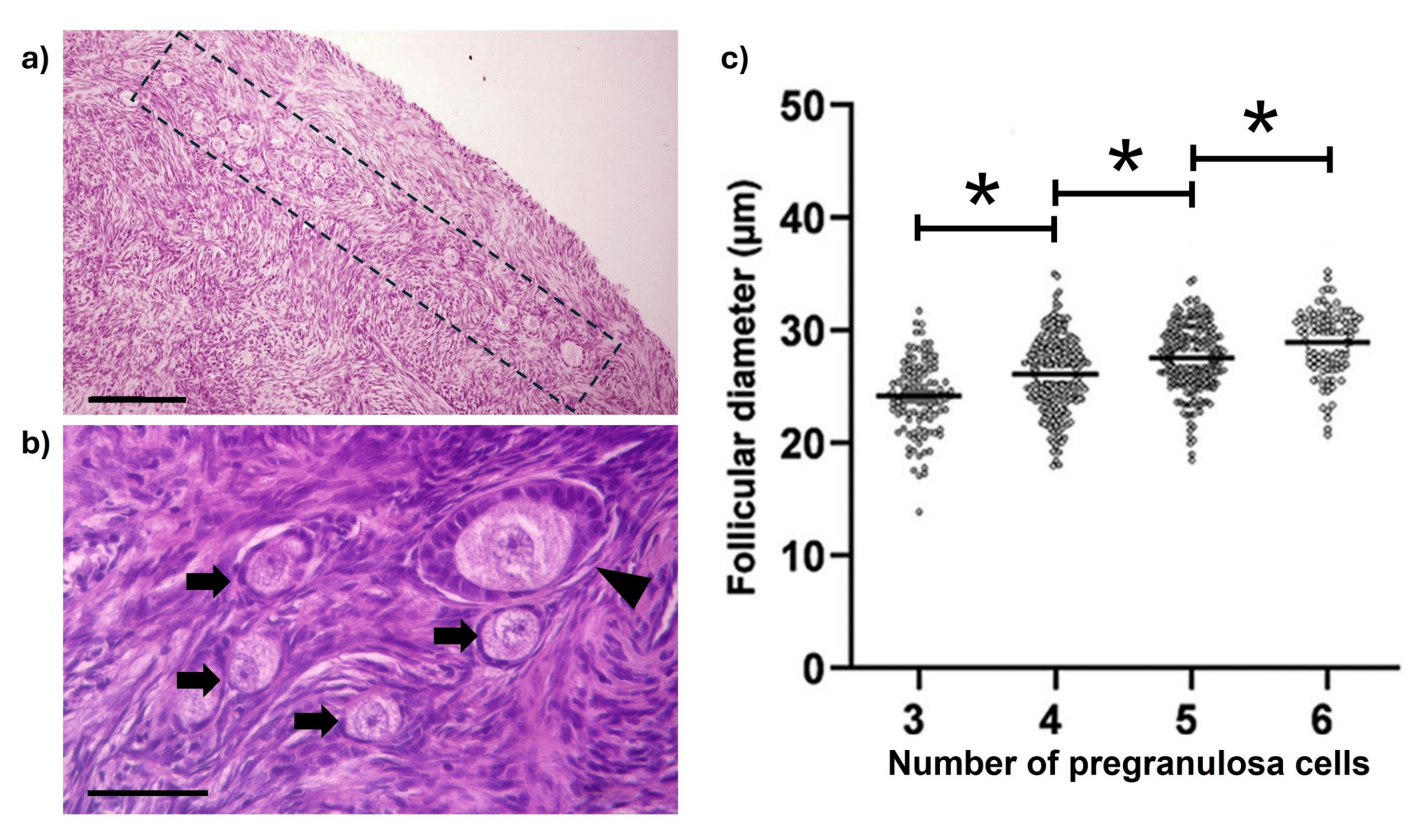
Primordial follicle characterization. Characteristics of primordial follicles in the ovarian cortex of CS sheep: a) Histological appearance of the peripheral region of the ovarian cortex. The dotted line indicates the approximate location of the primordial follicle population. Scale bar: 100 μm. b) Higher magnification of the boxed area in (a). Several primordial follicles (arrows) and a growing follicle (arrowhead) are visible. Scale bar: 50 μm. c) Graph showing the relationship between follicular diameter and the number of associated pre-granulosa cells. Asterisks indicate statistically significant differences between groups (p < 0.05). Data were analyzed using ANOVA.

The population of primordial follicles was located at a depth of 124.3 ± 3.57 µm of the ovary surface (Figure 3a); 76% of follicles were classified as primordial and characterized by the presence of an evident oocyte with nucleus and nucleolus, surrounded by three to six flat cells (Figure 3b). 83% of primordial follicles were surrounded by more than four cells; follicular diameter increased by the number of associated follicular cells (Figure 3c). The remaining follicles (24%) showed a mix of flat and cubic cells (transitional follicles) or were surrounded by cubic follicular cells (primary follicles). No other type of follicles (secondary or tertiary) was observed in the layers or strips dissected.

**Figure 4:**
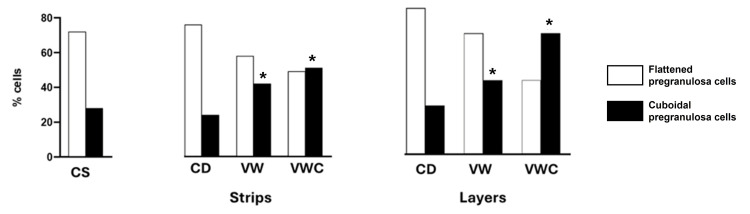
Resting and activated primordial follicles during the vitrification procedure. Percentage of resting follicles (with only flattened pregranulosa cells) and activated follicles (with at least one cuboidal pregranulosa cell) in the ovarian cortex of controls at the slaughterhouse (CS), strips or layers after dissection (CD), vitrification and warming (VW), or after culture (VWC). (*p < 0.05 vs. CS). Data were analyzed using the Chi-square test.

Processing of strips of ovarian cortex took two to three minutes more than the layers. No changes in the percentage of follicles surrounded by flat cells were detected immediately after the preparation of strips or layers compared to tissue fixed at the slaughterhouse. The number of primordial follicles found in a piece of tissue was variable (0 to 120), regardless of the dissection type. Approximately 5% of the histological sections obtained lacked primordial follicles. Samples examined after vitrification (VW) or after culture (VWC) showed a significant decrease in the percentage of primordial follicles and an increase in transition follicles showing at least one cubic follicular cell in both types of procedures: strips or layers (Figure 4).

Foxo3a immunolabeling

**Figure 5:**
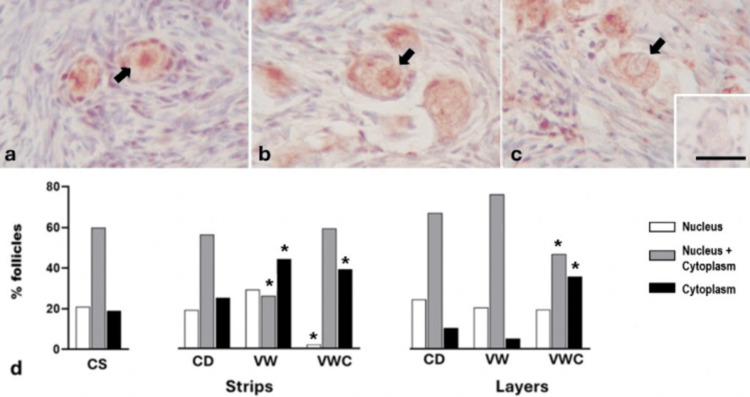
Representative images of Foxo3a immunohistochemistry. Primordial follicles (arrows) show immunoreactivity in the nucleus (a), both nucleus and cytoplasm (b), or cytoplasm only (c). Inset: negative control. The scale bar represents 25 μm in all images. (d) Percentage of follicles labeled with Foxo3a in control ovarian cortex (CS), after cutting into strips or layers (CD), following vitrification and warming (VW), or after vitrification, warming, and culture (VWC). *A significant difference compared to the corresponding category in the CS group (p < 0.05). Data were analyzed using the Chi-square test.

All follicles in strips and layers showed a Foxo3a signal. The label was observed in the nucleus of resting follicles (Figure 5a); in both the nucleus and cytoplasm of transitory follicles (Figure 5b); or exclusively in the cytoplasm of activated follicles (Figure 5c). The cortex of ovaries fixed at the slaughterhouse or the laboratory showed 20% of primordial follicles in the resting stage, nearly 60% in the process of activation and 20% in the active phase. The process of VW in tissue cut-in strips induced a significant reduction in the percentage of transitional follicles and an increase in activated follicles. After culture, only a small percentage of follicles remained in the resting stage since almost all were found in a transitional or activated stage. Tissue sectioned in layers subjected to VW procedures showed a higher percentage of follicles in the transitional stage, and when they were cultured, transitional follicles were reduced with an increase in the activated follicles (Figure 5d).

Cleaved caspase3 labeling

**Figure 6:**
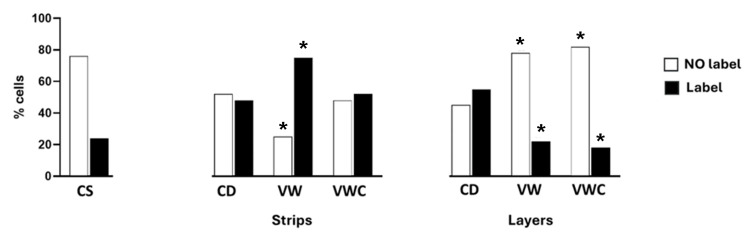
Cleaved caspase3 labeling. Percentage of follicles in the ovary cortex labeled with cleaved caspase3 in control (CS), after cutting (CD), exposed to vitrification and warming (VW), or vitrification, warming, and culture (VWC). *Significant difference with the corresponding category in the CS group (p < 0.05).  Data were compared using the Chi-square test.

Tissue fixed at slaughterhouse showed 20% of follicles immunoreactive for caspase; tissue cut in strips and subjected to WV or VWC significantly increased this percentage: follicles in tissue cut in layers showed a minor percentage of cleaved caspase3 positive label after VW or VCW (Figure 6).

Stromal tissue 

**Figure 7:**
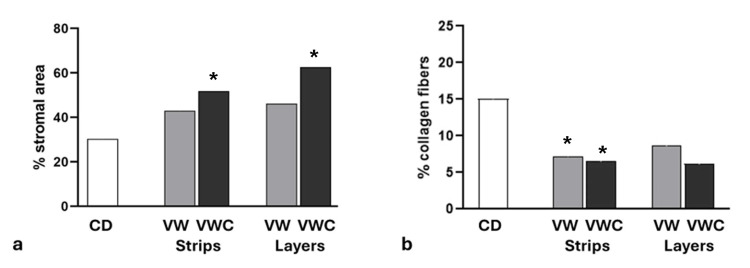
Stromal area during vitrification procedure. a) Percentage of the stromal area in the ovarian cortex after cutting into strips or layers and exposure to vitrification or vitrification followed by culture. b) Percentage of the stromal area occupied by collagen fibers in the ovarian cortex after cutting into strips or layers and exposure to vitrification or vitrification followed by culture. *Significant difference with the CD group (p < 0.05). Data were compared using the Chi-square test.

The connective tissue surrounding follicles in ovaries fixed after vitrification and culture was more diffuse than that fixed at the slaughterhouse. The quantification of the stromal area showed a significant increase in both samples (strips or layers) (Figure 7a). This effect correlated with a smaller proportion of collagen fibers per area in tissue cut in strips (Figure 7b).

## Discussion

Results in this communication show that manipulations carried out by the resection of the ovarian cortex impact the activation of primordial follicles and induce changes in the stromal compartment of tissue exposed to vitrification. These events could alter the oocyte viability and its final reproductive potential.

The vitrification of the ovarian cortex offers hope for restoring gonadal function in young women exposed to gonadotoxic effects provoked by chemo or radiotherapy [16]. The increasing instances of restoration of ovarian activity and pregnancy after post-grafting cryopreserved ovarian tissue are a testament to this [17,18]. Ovarian tissue vitrification as a fertility preservation technique is not considered experimental by the American Society of Reproductive Medicine [6]. While a single vitrification protocol has not been achieved, our study provides positive evidence that the transport of the ovary in saline solution and the presence of antibiotics is safe and does not produce significant effects on the viability of primordial follicles, at least during short transport times, as shown in diverse reports [19-21]. This paves the way for potential clinical applications and a brighter future in the oncofertility field.

An intriguing piece of information is the number of primordial follicles found in each of the fragments obtained after cutting the ovarian cortex. As our study shows, the number of primordial follicles found in sheep ovarian tissue was very variable in both strips and layers. Studies on human ovarian tissue have also shown a non-homogeneous distribution of primordial follicles [22,23]. This variability could help to explain the differences in results obtained using different types of dissection, including those informed in women, in terms of transplant efficiency. It seems crucial to gain a deeper understanding of the primordial follicle distribution in both animal models and women to optimize tissue dissection and outcomes after reimplantation.

Our results further support that handling during dissection and vitrification processes induces activation and initiates molecular pathways associated with cell death. For more than a decade, Gavish et al. [24] have shown that the duration of graft survival after transplantation of cryopreserved ovarian tissue is variable due to an intense activation and "burn-out" of dormant primordial follicles. Ostensibly, the mechanisms associated with the process of massive activation of primordial follicles include regulatory pathways such as the Hippo and Kit ligands, with both repressive and stimulatory signals disrupted by the fragmentation of the ovarian cortex [25]. These pathways play a crucial role in the activation and dormancy of primordial follicles. Furthermore, associated or added to the tissue response induced by dissection are the effects caused by the chemical substances used in the process of cryopreservation to prevent the formation of crystals but that, even in low concentrations, affect cell viability, such as dimethyl sulfoxide [26,27]. Leonel et al. [26] showed that cryoprotectants induce changes in the mitochondrial structure and chromatin condensation in both stromal and follicular cells. However, they are still widely used because of their significant benefits in preventing the formation of crystals that could cause cell rupture.

In this work, the fragmentation of ovarian tissue was performed using two models, one involving the scraping of adjacent tissue (strips) and the other obtained by superficial sections of the ovarian cortex (layers) with less force and time of execution than scraping. In both cases, the dissection of tissue induced an increase in the percentage of activation of primordial follicles. Since the population of dormant primordial follicles is the nonrenewable population that determines the reproductive lifespans of women, its activation limits the efficiency of the technique. Under normal physiological conditions, primordial follicle activation involves the morphological change of epithelial cells surrounding the oocyte from flat to cuboidal. Results from Engler et al. [28] have shown that morphological changes in cell shape are accompanied by modifications in gene expression and cellular differentiation capacity. However, the mechanisms that transduce the mechanical signal are not precise [11]. 

The distribution of Foxo3a immunolocalization in this study is consistent with the data shown by histological analysis, suggesting that the sharpest cut (in strips) causes greater and faster activation than the processing of layers. However, the culture and observation period would have to be extended to confirm these short-term results, highlighting the need for further research in this area and the urgency of understanding these mechanisms.

Other ovarian components that were affected by the manipulation were both the stromal area and the quantity of collagen fibers per area, which decreased after culture. Results published by Woodruff and Shea [5] further support the idea that the integrity of the ECM maintains follicles in the quiescent stage, limiting follicle expansion. It is plausible to infer that the diminution of contention by collagen fibers contributes to the activation of primordial follicles, which is a key factor in the efficiency of the technique.

To our knowledge, this is the first work that analyzes differences in tissue manipulation and its impact on the stromal compartment after vitrification. However, our work has limitations related to the time to culture and observation period. Culturing the tissue for a longer period would have been useful to evaluate the tissue vitality and would have given this study relevant information. There is still a need for deeper analyses to make conclusions about which is the best way of processing the tissue before vitrification to obtain better outcomes after thaw and reimplantation. 

## Conclusions

Ovarian tissue vitrification may be considered a standard method; however, there remain unanswered questions regarding tissue processing, cryopreservation techniques, and their impact on all compartments of the cryopreserved tissue. Continued investigation is needed into the role of the stromal compartment in the outcomes of ovarian tissue cryopreservation and reimplantation, as well as the development of new culture systems and xenografting methods.
